# Combination Effect of Engineered Endolysin EC340 With Antibiotics

**DOI:** 10.3389/fmicb.2022.821936

**Published:** 2022-02-15

**Authors:** Hye-Won Hong, Young Deuk Kim, Jaeyeon Jang, Min Soo Kim, Miryoung Song, Heejoon Myung

**Affiliations:** ^1^LyseNTech Co., Ltd., Seongnam-si, South Korea; ^2^Department of Bioscience and Biotechnology, Hankuk University of Foreign Studies, Yongin-si, South Korea; ^3^The Bacteriophage Bank of Korea, Hankuk University of Foreign Studies, Yongin-si, South Korea

**Keywords:** endolysin, bacteriophage, Gram-negative bacteria, antibacterial agent, cecropin A, colistin, synergy

## Abstract

Bacteriophage lysins, also known as endolysins or murein hydrolases, are hydrolytic enzymes produced by bacteriophages during the final stage of the lytic cycle to enable cleavage through the host’s cell wall, thus allowing the phages to burst out of their host bacteria after multiplication inside them. When applied externally to Gram-negative bacteria as recombinant proteins, lysins cannot easily reach the cell wall due to the presence of an outer membrane (OM). In this study, endolysin EC340 obtained from phage PBEC131 infecting *Escherichia coli* was engineered for improved OM permeability and increased activity against Gram-negative bacteria. The engineered endolysin, LNT113, was tested for potential synergistic effects with standard-of-care antibiotics. A synergistic effect was demonstrated with colistin, while an additive effect was seen with meropenem, tigecycline, chloramphenicol, azithromycin, and ciprofloxacin. Neither ceftazidime nor kanamycin showed any synergy or additive effects with the LNT113 endolysin. Moreover, synergy and additive effects could not be generalized by antibiotic class, OM traverse mechanism, molecular weight, or the bactericidal nature of each antibiotic tested.

## Introduction

The increase in multidrug-resistant (MDR) bacteria poses a major threat to morbidity and mortality worldwide. In particular, Gram-negative bacteria, such as carbapenem-resistant *Acinetobacter baumannii* and *Pseudomonas aeruginosa*, carbapenem-resistant and third-generation cephalosporin-resistant *Enterobacteriaceae*, are becoming a serious problem, especially in nosocomial infections (Luepke et al., 2017; Tacconelli et al., 2018).

The enzymatic activity of endolysin, produced by bacteriophages, degrades the peptidoglycan layer of host bacteria. In the late life cycle of phages, holin, a small membrane protein, forms pores in the bacterial cytoplasmic membrane, and when endolysin produced in the cytosol reaches this peptidoglycan layer, cell lysis results (Wang et al., 2000). Due to this enzymatic activity, endolysins with potential antibacterial activities have been reported (Schmelcher et al., 2012). Unlike with Gram-positive bacteria, in which the peptidoglycan cell wall is exposed, most extracellular endolysins are limited in their ability to degrade peptidoglycan due to the presence of an outer membrane (OM) in Gram-negative bacteria. That being said, some exceptions have been reported. LysSS has bactericidal activity in various MDR strains such as *A. baumannii* and *P. aeruginosa* (Kim et al., 2020). Bacteriophage lysin LysAM24 has antibacterial activity against various Gram-negative clinical bacteria and increased activity upon the addition of EDTA, an OM permeabilizer (Antonova et al., 2019). In addition to EDTA, the activity of *Salmonella* phage endolysin Lys68 was increased by using organic acids such as citric acid or malic acid as permeabilizers (Oliveira et al., 2014). Antimicrobial peptide fused to endolysin led to an increase in OM permeability, which in turn increased lytic activity. For example, artilysin increased the antibacterial activity by fusion of 29 amino acids of myeloid antimicrobial peptide to the N-terminus of KZ144 (Briers et al., 2014).

A synergy between endolysins and antibiotics has previously been reported. LysABP-01 endolysin from an *A. baumannii* phage resulted in increased growth inhibition and a synergistic effect in combination with colistin; however, combination with other antibiotics demonstrated no such growth inhibition (Thummeepak et al., 2016). ElyA1 endolysin with muralytic activity in various MDR strains exhibited increased activity when combined with colistin through minimum inhibitory concentration (MIC) and time-kill assay. This effect was observed in various strains of *A. baumannii* and *P. aeruginosa*, but not in *Klebsiella pneumoniae* (Blasco et al., 2020). The synergistic effect of antibiotics and endolysin was observed in Gram-positive as well as Gram-negative bacteria. Cpl-711, a chimeric endolysin, was confirmed to have a synergistic effect with various antibiotics in several MDR *S. pneumoniae* strains (Letrado et al., 2018). There were differences between antibiotics with synergism dependent on strains, but synergy was observed only with antibiotics belonging to the β-lactam family (Amoxicillin and Cefotaxime) in a time-kill assay. On the other hand, LysSS, an endolysin having activity against several MDR *A. baumannii* strains, showed only additive effects, and no synergy, in combination with colistin (Kim et al., 2020). Combination use of LysMK34 from phage MK34 infecting *A. baumannii* resulted in a 32-fold reduction in the MIC of colistin (Abdelkader et al., 2020). A synergistic antibacterial effect was also observed when an N-terminally cecropin A-fused LysMK34 and colistin were used in combination (Abdelkader et al., 2022).

In this study, we report on an engineered endolysin with an enhanced antibacterial activity against Gram-negative pathogens, as well as the observed synergistic effects when used in combination with standard-of-care antibiotics.

## Materials and Methods

### Bacterial Strains and Culture Conditions

The bacterial strains used in this study were obtained from the American Type Culture Collection (ATCC, United States), the Korean Collection for Type Cultures (KCTC, South Korea) and the Culture Collection of Antimicrobial Resistant Microbes (CCARM, South Korea). F strains and uropathogenic *Escherichia coli* (UPEC) strains were clinically isolated and were gifts kindly provided by Professor Kwan Soo Ko (Sungkyunkwan University School of Medicine). Adherent invasive *E. coli* (AIEC) collection (ECOR) strains were generously provided by Professor Christel Neut (Rahmouni et al., 2018). The MCR-1 positive *E. coli* FORC81 strain was kindly provided by Professor Sangryeol Ryu (Seoul National University) (Kim et al., 2019). All strains used in this work were grown in LB (lysogeny broth) or CAA medium (5 g/l casamino acids, 5.2 mM K_2_HPO_4_, and 1 mM MgSO_4_) at 37°C.

### Bacteriophages and Endolysins

Bacteriophage PBEC131 infecting *E. coli* was obtained from the Bacteriophage Bank of Korea.^1^ Gene expressing putative endolysin (GenBank accession no. OL415943) was obtained from bacteriophage PBEC131 and the putative lysozyme-like superfamily domain was found through BLASTp.

### Molecular Cloning

**FIGURE 1:**
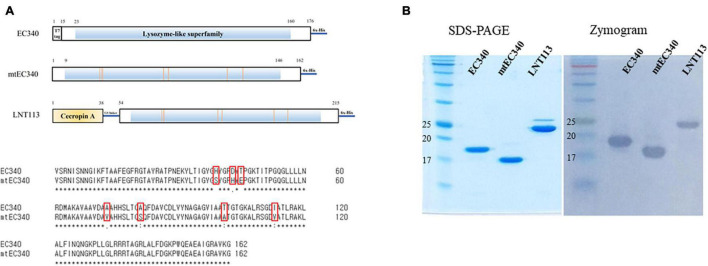
Organization of endolysins used in this study and their enzymatic activities. **(A)** Domain structure of EC340, mtEC340, and LNT113. It has a predicted lysozyme domain without a cell-wall binding domain (CBD). EC340 from *E. coli* phage PBEC131 was subjected to several substitutions of amino acids (mtEC340). The locations of substituted amino acids are shown. mtEC340 had cecropin A fused to its N-terminus and was named LNT113. **(B)** Analysis of purified endolysins, EC340 (19.5 kDa), mtEC340 (18.2 kDa), and LNT113 (23.2 kDa) on SDS-PAGE and subsequent zymogram assay.

The gene (GenBank accession no. OL415943) encoding putative endolysin from bacteriophage PBEC131 was cloned into pET21a+ (Novagen, United States) using BamHI and XhoI restriction sites, and named EC340. A mutant EC340 was generated by substituting seven amino acids (Figure 1A). LNT113 was constructed by fusing the antimicrobial peptide cecropin A (NCBI PRF 0708214A) to the 5′ end of mutant EC340 with the (GGGGS)_3_ linker. All constructs have a C-terminal hexa-histidine tag for affinity purification. Also, the gene encoding enhanced green fluorescent protein (EGFP; GenBank accession no. AAB02576.1) was cloned with the gene encoding cecropin A (CecA) to its 5′ end into pET21a+ vector.

### Recombinant Protein Purification

Endolysin expression vectors were introduced into *E. coli* BL21 (DE3) Star (Invitrogen, United States) for the expression of protein. Plasmid harboring gene encoding CecA-fused EGFP was introduced into *E. coli* BL21 (DE3) pLysS (Novagen) for expression. The cells were grown in LB broth to an exponential phase (OD_600_ = 0.4–0.5) and then induced with isopropyl β-D-1-thiogalactopyranoside (IPTG; Duchefa, Netherlands) at a concentration of 0.5 mM at 25°C for 5 h. The bacterial cells were harvested by centrifugation and the pellets washed with phosphate-buffered saline (PBS). The cells were resuspended in lysis buffer (20 mM Tris–HCl, pH 7.5, 300 mM NaCl, and 20 mM imidazole) and disrupted by sonication (Sonics, United States) for 5 min. The supernatant was obtained by centrifugation at 15,000 × *g* for 30 min from cell lysate. The extracts were loaded onto an Ni-NTA affinity chromatography column using FPLC (AKTA go, Cytiva, United Kingdom). The proteins were eluted using a linear gradient from 20 to 500 mM of imidazole. The proteins were then loaded onto a HiTrap SP HP column (Cytiva) for cation exchange chromatography and eluted with a linear gradient of NaCl from 0 to 1 M in 20 mM Tris–HCl, pH 7.5. The proteins were then dialyzed in the storage buffer [20 mM Tris–HCl, pH 7.5, 150 mM NaCl buffer (pH 7.5)].

### Zymogram Assay

Zymogram assay was performed based on a previously described method with some modification (Khakhum et al., 2016). Briefly, an overnight culture of *E. coli* ATCC 8739 was harvested and washed once with PBS and then harvested by centrifugation at 4,000 × *g* for 15 min. The pellet was then resuspended in 3 ml of deionized water. Cells were autoclaved and added to a 15% SDS-PAGE gel before polymerization. Then, 3 μg of purified endolysins (EC340, mutant EC340, or LNT113) were mixed with 2× sample buffer (0.5 mM Tris–HCl, pH 6.8, 20% glycerol, 0.2% bromophenol blue), and loaded into SDS-PAGE. After electrophoresis, the gel was washed with deionized water for 1 h and incubated in reaction buffer (1% Triton X-100, 20 mM Tris–HCl, pH 7.5) at room temperature. The enzymatic activity of the endolysins was observed by clear zones on gel containing *E. coli* lysate.

### Antibacterial Activity Assay

The antibacterial activity of the purified protein was tested against *E. coli* and several *K. pneumoniae* strains. Bacteria were grown to the exponential phase (OD_600_ = 0.5), and harvested by centrifugation at 12,000 × *g* for 3 min. Then, the pellets were washed with reaction buffer (20 mM Tris–HCl, pH 7.5) and diluted in the buffer to approximately 10^6^ cells/ml. Next, 100 μl of the bacterial suspension was mixed with 100 μl of the purified endolysin in reaction buffer and incubated at 37°C for 2 h. Finally, the mixtures were diluted with PBS and loaded on LB plates. After overnight incubation at 37°C, bacterial colonies were counted. All assays were performed in triplicate. The same reaction was also carried out in 20 mM HEPES-NaOH, 150 mM NaCl (pH 7.4) for the selected target bacteria, to exclude possible intrinsic antibacterial activity of Tris and endolysin’s dependence on turgor pressure (Supplementary Figure 2).

### 1-*N*-Phenylnaphthylamine Uptake Assay

An 1-*N*-Phenylnaphthylamine (NPN) uptake assay was performed by methods previously described (Helander and Mattila-Sandholm, 2000). Briefly, *E. coli* ATCC 8739 grown to the exponential phase (OD_600_ = 0.4) was washed and resuspended in buffer (5 mM HEPES, pH 7.2) to approximately 10^8^ cells/ml. Fifty microliters of 40 μM NPN (Sigma-Aldrich) was mixed with 50 μl of purified endolysin or cecropin A (AbClon, South Korea) to a final concentration of 2 μM on 96-well black plates. OM permeabilizers, 1 mM EDTA (Duchefa) and 2 μM Polymyxin B (Sigma-Aldrich) were used as positive controls. Then, 100 ml of cell suspension was added to each well and incubated at 37°C for 5 min. Buffer only, buffer + NPN, bacterial suspension + buffer, and bacterial suspension + buffer + NPN, were used as negative controls. Fluorescence was measured at excitation (350 nm) and emission (420 nm) with a microplate reader (SpectraMax iD3, Molecular Devices, United States). The NPN uptake factor was calculated by dividing the fluorescence value after background subtraction (fluorescence value in the cell mixture with the sample subtracted by the value without NPN) by the fluorescence value of the cell suspension with the buffer subtracted from that of buffer without NPN.

### Hemolysis Assay

Sheep red blood cells (RBCs) were used for the *in vitro* hemolytic activity test. One milliliter of RBCs (MB Cell, South Korea) was diluted with 9 ml of PBS. Then, 180 μl of RBC solution was added to 20 μl of LNT113 (final concentration, 2–128 μg/ml), PBS (negative control), or 0.1% Triton X-100 (positive control) and incubated at 37°C for 30 min. The mixtures were centrifuged at 500 × *g* for 5 min, and the supernatants were transferred to 96-well microplates. Absorbance was measured at 570 nm. The hemolysis rate was calculated using the following equation:


%Hemolysis={[Abs⁢(Sample)-Abs⁢(negativecontrol)][Abs⁢(positivecontrl)-Abs⁢(negativecontrol)]}⁢x⁢ 100.

### *In vitro* Cytotoxicity Assay

A human hepatocellular carcinoma cell line, Huh7, was used for the cytotoxicity assay of LNT113. First, 1 × 10^4^ Huh7 cells were seeded in 96-well plates. After 24 h, LNT113 (final concentration, 125 or 250 μg/ml), 1% Triton X-100 (positive control) or PBS (negative control) was added to the cells and incubated for 24 or 48 h. Then, 10 μl of tetrazolium salt solution in a WST-8 Cell Viability Assay Kit (Dyne Bio, South Korea) was added to each well. After 1 h of incubation at 37°C in a 5% CO_2_ incubator, the production of formazan was measured at 450 nm.

### Measuring Minimum Inhibitory Concentration and Minimum Bactericidal Concentration

The MICs of LNT113 or antibiotics were determined using a modification of the broth microdilution method in 96-well, round-bottomed, microplates according to a previously described method (Heselpoth et al., 2019). The exponentially grown cells were diluted to a concentration of 10^6^ CFU/ml in CAA medium and then incubated with recombinant LNT113 (1–64 μg/ml) or antibiotics at 35°C for 20 h. The MIC values were determined as the lowest antibacterial concentration that completely inhibited bacterial growth. The minimum bactericidal concentration (MBC) was determined by spotting 10 μl of the mixture from each well of the MIC test plate on LB agar. The MBC was defined as the lowest concentration of antimicrobial agent with no growth on the plate. All assays were performed in duplicate.

### Checkerboard Assay

A checkerboard assay was performed utilizing the serial dilution method as described previously (Thummeepak et al., 2016). LNT113 was diluted twofold vertically and antibiotics were serially diluted horizontally in 96-well microplates. To each well was added bacteria at concentrations of 10^6^ CFU/ml in CAA media. After incubation at 35°C for 20 h, MICs were visually confirmed for cells that did not grow. The fractional inhibitory concentration (FIC) for LNT113 or antibiotics was calculated by dividing the MIC of two drugs in combination with the MIC of each drug alone. The FIC index (FICI), the sum of the FICs of each of the drugs, was used to confirm the interaction of the two drugs. The FICI was considered to be as follows: synergistic effect ≤0.5, additive >0.5 to ≤1, indifference >1.0 to ≤2, and antagonism >2.

### Statistical Analysis

Prism version 9 (GraphPad software) was used for all statistical analysis. For *in vitro* studies, all experiments were carried out in triplicate and the results are given as means ± standard error of the means (SEM). Two-way ANOVA with Tukey’s multiple comparison test was used to compare the differences between each dataset.

## Results

### Endolysin From Bacteriophage PBEC131 and Its Engineering

**FIGURE 2:**
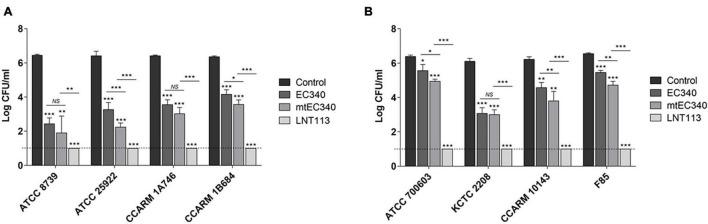
Antibacterial activities of endolysins. Antibacterial activity of endolysins (2 μM) against *E. coli* strains **(A)** and *K. pneumoniae* strains **(B)** in 20 mM Tris–HCl buffer (pH 7.5) for 2 h. Tris buffer was used as a negative control. Dotted line denotes the detection limit. In addition to *t*-tests, two-way ANOVA was performed and is shown as horizontal bars above vertical bars (*p < 0.05, **p < 0.01, ***p < 0.001).

An ORF consisting of 162 amino acids from phage PBEC131 was annotated as a putative endolysin. This endolysin, named EC340 (GenBank accession no. OL415943), has a phage-related lysozyme (muramidase) domain (pfam00959) (Figure 1A). This gene was cloned into an expression vector and expressed, and the enzymatic activity was observed by performing a zymogram assay with the purified endolysin (Figure 1B). To improve the activity of the endolysin, BLASTp was performed with 8 proteins having highly similar sequences to EC340 (GenBank accession nos. QNO11705.1, QNO11629.1, QNO11777.1, QBJ02951.1, HAM5207786.1, YP_009168880.1, QIG59335.1, and YP_009113200.1) (Supplementary Figure 1). We also ran a computer-aided modeling of the protein at https://swissmodel.expasy.org/. Specifically, we considered that changes in externally exposed amino acids H39S, A73V, T101A, and I113V, might increase the hydrophobic nature of the protein, resulting in a better transmembrane passage. Changes D43H and T45E are those in an externally exposed hinge region, where switches in charged amino acids may result in a sterically more stable nature for the region. The change in A81S, located in the third helical region of the protein, might not have induced a notable change in the characteristics of the protein. Mutations were introduced at these seven amino acid positions within the enzymatic activity domain of EC340 (Figure 1), leading to a slight change in GRAVY. The resulting mutant, named mtEC340, exhibited increased antibacterial activity by up to 1 log (Figure 2) when tested against various strains of *E. coli* or *K. pneumoniae* including type strains, drug-resistant strains (the resistance profile is shown in Supplementary Table 1), and clinically isolated strains. Higher antibacterial efficacy for the endolysins was observed in *E. coli* than in *K. pneumoniae*. This is consistent with a previous report where it was noted that the thickness of the capsule in *K. pneumoniae* was more than 16 times that seen in *E. coli* (Amako et al., 1988). Furthermore, one of the *K. pneumoniae* strains used in this experiment, ATCC700603, is known to have a capsular structure (Formosa et al., 2015). Thick capsules likely hinder the access of endolysins to peptidoglycan cell walls. *K. pneumoniae* capsular polysaccharide was reported to mediate resistance to antimicrobial peptides such as polymyxin and lactoferrin (Campos et al., 2004). To further increase the activity, endolysin LNT113 was constructed by fusion of a 37 amino-acid sequence of cecropin A, an antimicrobial peptide, to the N-terminus of mtEC340. This demonstrated up to a 4-log increase in activity when compared to mtEC340. No viable bacterial cells were detected after 2 h of incubation for all strains tested. Since the antibacterial activity of endolysins potentially depends on turgor pressure (Abdelkader et al., 2020), and Tris itself might act as an OM permeabilizer (Hancock and Wong, 1984), we performed additional antibacterial assays under differing physiological conditions; in Tris buffer containing 150 mM NaCl or in Hepes buffer containing 150 mM NaCl (Supplementary Figure 2). Unexpectedly, antibacterial activities were higher in the Hepes buffer than in the Tris buffer without NaCl. But, the addition of NaCl rendered EC340 and mtEC340 almost inactive. However, the negative effect of the presence of salt was greatly decreased when cecropin A-fused endolysin (LNT113) was used as reported previously (Abdelkader et al., 2022).

### Enhanced Cell Permeability of LNT113 Led to Enhanced Antibacterial Efficacy

**FIGURE 3:**
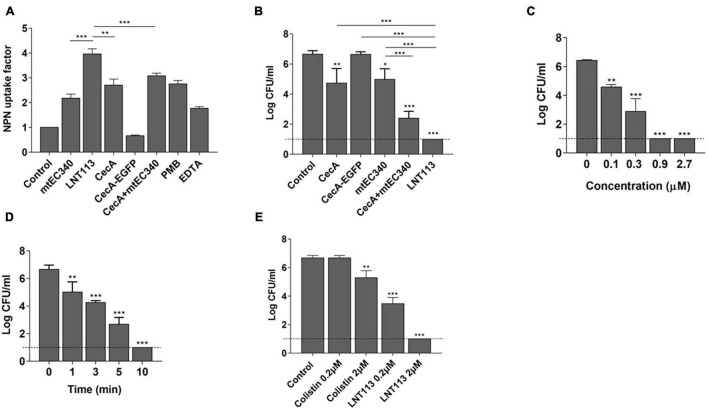
Cell penetration and antimicrobial activity of LNT113. **(A)** The outer membrane permeability of the Gram-negative *E. coli* ATCC 8739 was determined by NPN assays. CecA-EGFP; cecropin A fused to EGFP, cecA+mtEC340; cecropin A and mtEC340 were separately added to the mixture at 2 μM each, PMB; polymyxin B. **(B)** LNT113 showed an enhanced antibacterial activity against *E. coli* compared to Cecropin A and/or mtEC340. All were treated to a final concentration of 0.5 mM. **(C)** Antibacterial activities of different concentrations of LNT113 (0.1, 0.3, 0.9, or 2.7 μM) against *E. coli* ATCC 8739. **(D)** Decrease in bacterial viability after the addition of endolysin (0.5 μM) at different time lengths. Dashed lines show the detection limit. **(E)** Antibacterial activity of LNT113 against MCR-1 positive *E. coli* FORC81. The cells were treated with LNT113 or colistin at concentrations of 0.2 or 2 μM for 2 h. Dotted line denotes the detection limit. Asterisks represent statistical differences compared to the control (*p < 0.05, **p < 0.01, ***p < 0.001).

Generally, externally administered endolysin has difficulty reaching the peptidoglycan layer due to the presence of an OM in Gram-negative bacteria. Fusion with cecropin A interacting with the OM should increase membrane penetration. We compared the membrane-penetrating ability of several constructs containing cecropin A and/or the endolysin (Figure 3A) through NPN assay. A 1.8-fold increase in membrane penetration was observed when cells which were treated with cecropin A-fused LNT113 as compared to mtEC340. This was also 1.3-fold higher than with cecropin A alone. Also, membrane permeability was increased when mtEC340 and cecropin A were treated in combination. Conversely, cecropin A fused to EGFP demonstrated a limited ability to penetrate the membrane when compared to LNT113, suggesting the partnering of an AMP with an endolysin which demonstrates a robust ability to penetrate the membranes is required to achieve an additive effect. The bactericidal efficacy of each construct shown in Figure 3B was proportional to the ability to permeate membranes, as shown in Figure 3A. *In vitro*, LNT113 exhibited antibacterial activity in dose- and time-dependent manners (Figures 3C,D). *E. coli* FORC81, a colistin-resistant strain, was tested for susceptibility to LNT113 (Figure 3E). At a concentration of 2 μM, a 1.2-log reduction in colistin-treated cells was observed, while LNT113, at the same concentration, managed to decrease the quantity of bacterial cells below the detection limit.

### Determination of Minimum Inhibitory Concentrations

*Escherichia coli* has been associated with a variety of diseases in humans and animals. Pathogenic *E. coli* causes diseases by colonizing various sites in the human body, such as the urinary tract, kidneys, and the bloodstream (Croxen and Finlay, 2010). We confirmed the antibacterial activity of LNT113 in various *E. coli* strains through determination of MIC (Table 1). Target strains included type strains, drug-resistant strains (CCARM and FORC81), clinically isolated strains (F), uropathogenic strains (UPEC), and adherent invasive strains (ECOR). The MICs were shown to be 4–64 μg/ml in most strains (1 mM of LNT113 is 23.2 mg/ml, and 1 mg/ml of LNT113 is 0.0431 mM). MICs for endolysins EC340 or mtEC340 were >128 μg/ml (Supplementary Table 2). In addition, MBCs were the same as MIC, suggesting the mode of action was bactericidal. The MICs of LNT113 were confirmed against various Gram-negative bacteria such as *A. baumannii, P. aeruginosa*, and *K. pneumoniae* (Table 2). The strains included type strains, clinically isolated strains, and drug-resistant strains. MICs of antibiotics for the drug resistance strains used are shown in Supplementary Table 1. MICs were at the range of 4–32 μg/ml. For *Salmonella typhimurium* and *Salmonella enteritidis*, MICs were >64 μg/ml (data not shown).

**TABLE 1:** Minimum inhibitory concentrations and minimum bactericidal concentrations of LNT113 against various *E. coli* strains.

Strains	MIC	MBC	Strains	MIC	MBC	Strains	MIC	MBC
ATCC 8739	8	8	UPEC 90	64	64	ECOR 1	8	8
ATCC 25922	64	64	UPEC 3038	16	16	ECOR 2	8	8
ATCC 51739	8	8	UPEC 3042	16	16	ECOR 9	4	4
CCARM 1A746	4	4	UPEC 3051	16	16	ECOR 15	32	32
CCARM 1G490	16	16	UPEC 3150	8	8	ECOR 35	16	16
F485	4	4	UPEC 3151	64	64	ECOR 36	>64	>64
F524	8	8	UPEC 3163	16	16	ECOR 43	16	16
F576	4	4	UPEC 3164	32	32	ECOR 45	4	4
F716	>64	>64	UPEC 3168	8	8	ECOR 52	64	>64
F852	8	16	UPEC 3181	8	8	ECOR 69	>64	>64
FORC81	8	8						

*MICs, minimal inhibitory concentrations; MBCs, minimum bactericidal concentrations.*

**TABLE 2:** Minimum inhibitory concentrations (μg/ml) of LNT113 against various Gram-negative bacteria.

*Acinetobacter baumannii*		*Pseudomonas aeruginosa*		*Klebsiella pneumoniae*		*Klebsiella aerogenes*	
Strains	MIC	Strains	MIC	Strains	MIC	Strains	MIC
ATCC 19606	8	PAO1	4	ATCC 700603	16	CCARM 16006	16
ATCC 17978	16	ATCC 15522	8	KCTC 2208	8	CCARM 16008	8
CCARM 12001	8	F102	16	CCARM 10143	4	CCARM 16010	8
F4	4	F125	4	F104	16	F276	8
F65	8	F141	16	F118	16	***Enterobacte cloacae***	
F66	8	F171	32	F144	16	ATCC 13047	16
F67	8	F388	32			CCARM 0252	4
						CCARM 16003	8

### Cytotoxicity and Hemolytic Activity of LNT113

**FIGURE 4:**
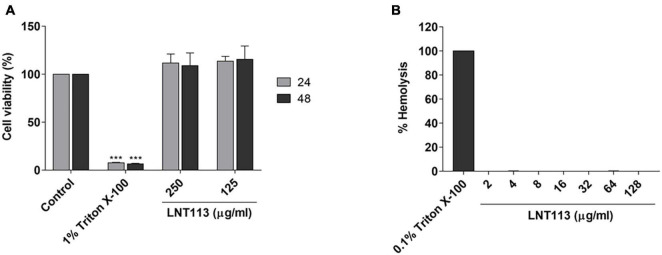
Cytotoxicity and hemolytic activity of LNT113. **(A)** Cytotoxicity assay of LNT113 in a Huh7 cell line. Cells were incubated with different concentrations of LNT113 for 24 or 48 h. Triton X-100 was used as the cytotoxicity control agent. **(B)** Hemolytic activity of LNT113. RBCs were incubated with PBS or LNT113 at 37°C for 1 h, and hemolysis was determined by measuring the absorbance of the supernatant at 570 nm. 0.1% of Triton X-100 was used as the hemolysis control agent. Statistical difference compared to control ***P < 0.001.

To determine the cytotoxicity of LNT113, we performed a WST-1 assay against human liver carcinoma cell line Huh7. No decrease in cell viability was observed by treating with LNT113 at 250 or 125 μg/ml for 48 h (Figure 4A). To determine hemolytic activity, LNT113 was added in RBCs at concentrations from 2 to 128 μg/ml. No hemolytic activity of LNT113 was observed (Figure 4B).

### Synergistic Effect of LNT113 and Various Antibiotics

**FIGURE 5:**
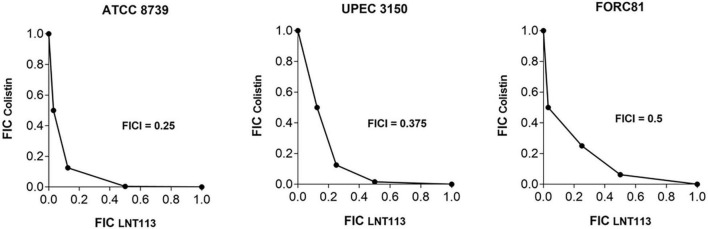
Synergistic effect of LNT113 and colistin against three different *E. coli* strains. The isobologram of LNT113 and colistin in *E. coli*. FIC indexes (FICIs) represent the sum of Fractional Inhibitory Concentrations (FICs) of LNT113 and colistin.

A checkerboard assay was performed to determine the effect of combining LNT113 with a variety of antibiotics. First, the combination effect between LNT113 and colistin was observed in *E. coli* strains by determining the FICI. The results of the checkerboard assay, represented by isobologram, including the plotting of FICs of LNT113 and colistin, can be seen in Figure 5. When both colistin and LNT113 were treated in *E. coli* ATCC 8739, the sum of the two values (FICI) was 0.25. This indicates a synergistic effect of the two agents. For UPEC 3150 strain, the FICI was 0.375, also demonstrating a synergistic effect. For *E. coli* FORC81, the FICI was 0.5, suggesting another synergistic effect. It is noteworthy that in colistin-resistant *E. coli* FORC81, the MIC of colistin decreased from 16 to 1 μg/ml when treated in combination with 4 μg/ml of LNT113 (Supplementary Table 2). Any synergistic effect between LNT113 and eight different antibiotics was checked against five different *E. coli* strains by determining the FICI (Table 3). For 4 out of 5 strains, a synergy was observed with colistin, while in all other combinations additive or indifferent effects were observed. We also observed that in the presence of endolysins EC340 or mtEC340, for which the MICs were >128 μg/ml, the MIC of colistin decreased notably (Supplementary Table 2).

**TABLE 3:** Fractional Inhibitory Concentration Index (FICI) of LNT113 with various antibiotics.

Antibiotics	*E. coli* strains				
	ATCC 8739	ATCC 51739	UPEC 3150	CCARM 1A746	FORC81
Colistin	0.25	0.5	0.38	0.63	0.5
Ceftazidime	2	2	1	2	1
Meropenem	0.63	0.75	1	1	2
Kanamycin	2	1	1	n.d.*	1
Tigecycline	2	0.63	0.63	2	2
Chloramphenicol	1	0.75	0.75	n.d.*	2
Azithromycin	1	0.75	0.75	1	2
Ciprofloxacin	0.75	0.75	0.75	1	0.56

*Not determined.

*FICI: ≤0.5, synergy; >0.5 to ≤1.0, additive; >1.0 to ≤2, indifference; >2.0, antagonism.*

## Discussion

Cecropin A, isolated from the hemolymph of *Hyalophora cecropia*, is a 37 amino-acid peptide with an amphipathic alpha-helix structure exhibiting antibacterial activity via interaction with the OM of Gram-negative bacteria (Hultmark et al., 1980; Holak et al., 1988; Steiner et al., 1988). Fusion of cecropin A to mtEC340 led to an increased cell membrane permeability, by 1.8 times, and an increased antibacterial property, by 2–4 logs, in this study. Cecropin A is reported to induce membrane disruption (Silva et al., 2018), but a generally agreed mode of action is yet to be elucidated. It is proposed that cecropin A crosses the OM by self-promoted uptake, causing distortions of the membrane (Scott et al., 1999). The distortion of the OM may facilitate uptake of cecropin-fused endolysin as well as cecropin itself, leading to a much higher antibacterial efficacy of the fusion protein, as demonstrated in this study.

Since the 1990s, colistin has been used as a last-resort treatment against the rapid increase of MDR pathogens (Kaye et al., 2016; El-Sayed Ahmed et al., 2020). LNT113 was confirmed to have a synergistic effect with colistin in checkerboard assays. Colistin directly interacts with lipid A of lipopolysaccharide (LPS), and has an important role in antibacterial activity by forming LPS-colistin clusters in synthetic LPS/phospholipid bilayers (Khadka et al., 2018). Mutation of a gene involved in lipid A biosynthesis produced LPS-deficient bacteria, leading to colistin resistance (Moffatt et al., 2010). Another resistance mechanism involves MCR-1, a member of the phosphoethanolamine transferase enzyme family, leading to the addition of phosphoethanolamine to lipid A, hindering the attachment of colistin (Liu et al., 2016). This interaction of colistin and lipid A may enable a more efficient uptake of the endolysin, resulting in a higher antibacterial efficacy of the fusion protein. No synergistic effect between LNT113 and antibiotics other than colistin was observed. Additive effects also differed depending on target strains, which was consistent with a previous report (Letrado et al., 2018). These differences were independent of antibiotic susceptibility. The MDR strain CCARM 1A746 (Supplementary Table 1) showed a higher FICI value, maybe due to the presence of multiple resistance mechanisms which inhibited the antibacterial effect of LNT113 in more than one way. No generalization of combination effects in accordance with different classes of antibiotics was possible. For example, ceftazidime, one of the two β-lactam antibiotics tested, possessed little additive effect with LNT113, while the other β-lactam antibiotic, meropenem, exhibited some additive effect. Based on mechanisms of OM traverse, macrolides such as azithromycin, and aminoglycosides such as kanamycin are those using lipid-mediated pathways, while β-lactams use porin-mediated diffusion (Benz, 1988; Nikaido, 2003). Again, no consistent pattern was observed among the groupings in this study. In addition, the molecular weight of each tested antibiotic or its bacteriostatic or bactericidal mechanism of action led to no consistent pattern, either.

## Conclusion

In summary, we produced an engineered endolysin LNT113 and confirmed its antibacterial efficacy *in vitro*. As it harbors a synergistic effect with colistin, and some additive effects with standard-of-care antibiotics, this endolysin could be used as a novel modality to overcome current antibiotic resistance issues.

## Data Availability Statement

The original contributions presented in the study are included in the article/Supplementary Material, further inquiries can be directed to the corresponding author/s.

## Author Contributions

HM, MS, and MK conceived and designed the experiments. H-WH, YK, and JJ performed the experiments and generated the data. HM wrote the manuscript. All authors contributed to the article and approved the submitted version.

## Conflict of Interest

H-WH, YK, JJ, MK, and HM were employed by LyseNTech Co., Ltd. The remaining author declares that the research was conducted in the absence of any commercial or financial relationships that could be construed as a potential conflict of interest.

## Publisher’s Note

All claims expressed in this article are solely those of the authors and do not necessarily represent those of their affiliated organizations, or those of the publisher, the editors and the reviewers. Any product that may be evaluated in this article, or claim that may be made by its manufacturer, is not guaranteed or endorsed by the publisher.

## Footnotes

1
www.phagebank.or.kr

## Supplementary Material

Supplementary Figure 1 Amino acid sequence alignment (CLUSTAL omega) of putative endolysins used in site-directed mutagenesis of EC340.

Supplementary Figure 2 Effect of turgor pressure and Tris in antibacterial activities of endolysins. Antibacterial activity of endolysins against *E. coli* ATCC8739 **(A)** and *K. pneumoniae* KCTC2208 **(B)**. Exponentially grown bacterial cells were incubated with 2 μM of endolysins in two different buffers (20 mM Tris–HCl, pH 7.5 or 20 mM HEPES, pH7.5) in the presence or absence of 150 mM NaCl for 2 h at 37°C. Dotted line denotes the detection limit. In addition to *t*-tests, two-way ANOVA was performed and is shown as horizontal bars above vertical bars (**p* < 0.05, ***p* < 0.01, ****p* < 0.001).
