# Corrigendum: Combination effect of engineered endolysin EC340 with antibiotics

**DOI:** 10.3389/fmicb.2023.1281242

**Published:** 2023-08-31

**Authors:** Hye-Won Hong, Young Deuk Kim, Jaeyeon Jang, Min Soo Kim, Miryoung Song, Heejoon Myung

**Affiliations:** ^1^LyseNTech Co., Ltd., Seongnam-si, South Korea; ^2^Department of Bioscience and Biotechnology, Hankuk University of Foreign Studies, Yongin-si, South Korea; ^3^The Bacteriophage Bank of Korea, Hankuk University of Foreign Studies, Yongin-si, South Korea

**Keywords:** endolysin, bacteriophage, Gram-negative bacteria, antibacterial agent, cecropin A, colistin, synergy

A correction has been made to Funding. In the original article a grant was mistakenly omitted. The corrected Funding statement appears below.

