# Supplementary figures and images for: Combination Effect of Engineered Endolysin EC340 With Antibiotics
Source: Front Microbiol. 2022 Feb 15;13:821936. doi: 10.3389/fmicb.2022.821936 (PMC8886149; doi:10.3389/fmicb.2022.821936)

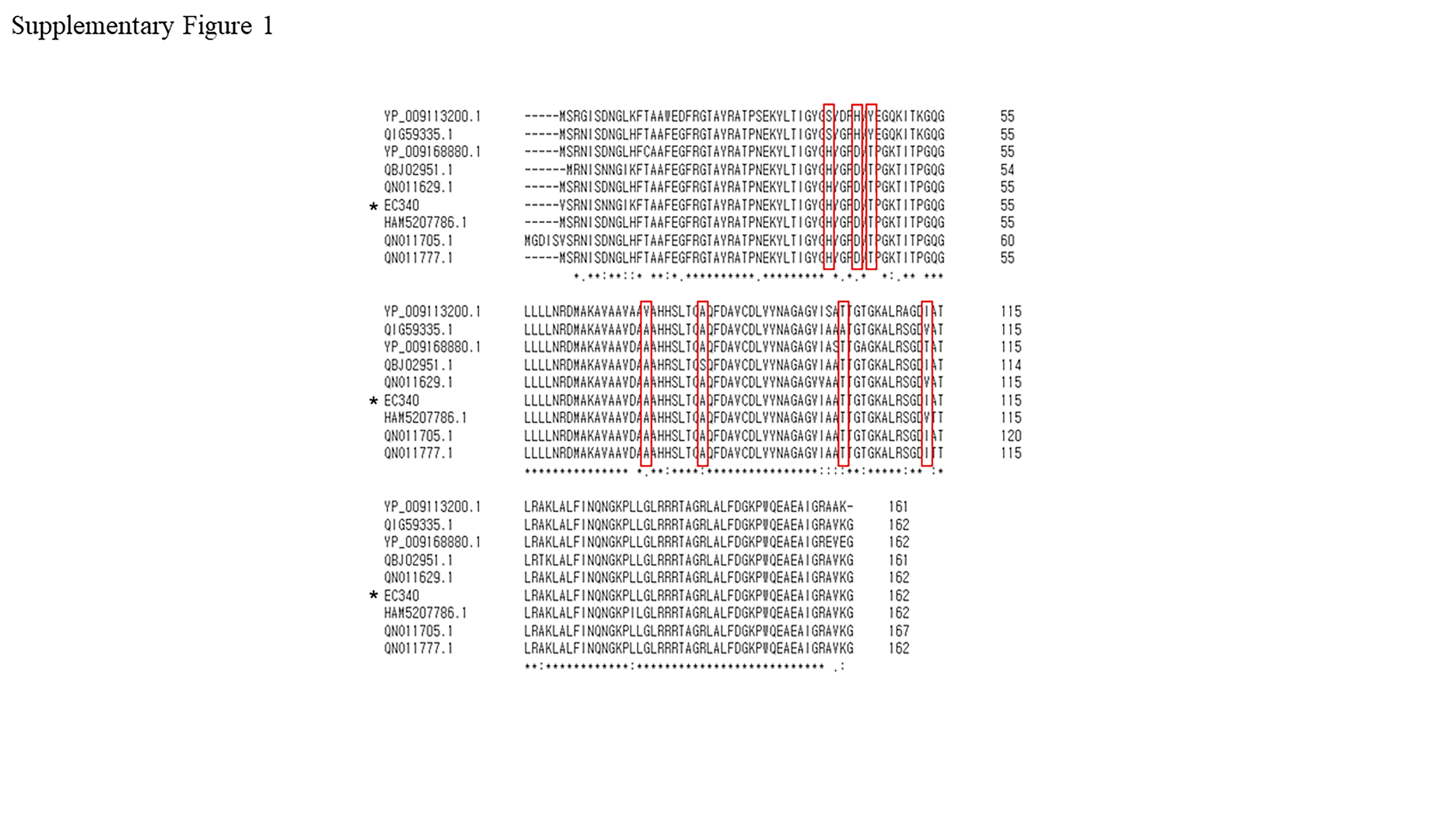

Supplement: Supplementary Figure 1 — Amino acid sequence alignment (CLUSTAL omega) of putative endolysins used in site-directed mutagenesis of EC340. [file Image_1.TIF]

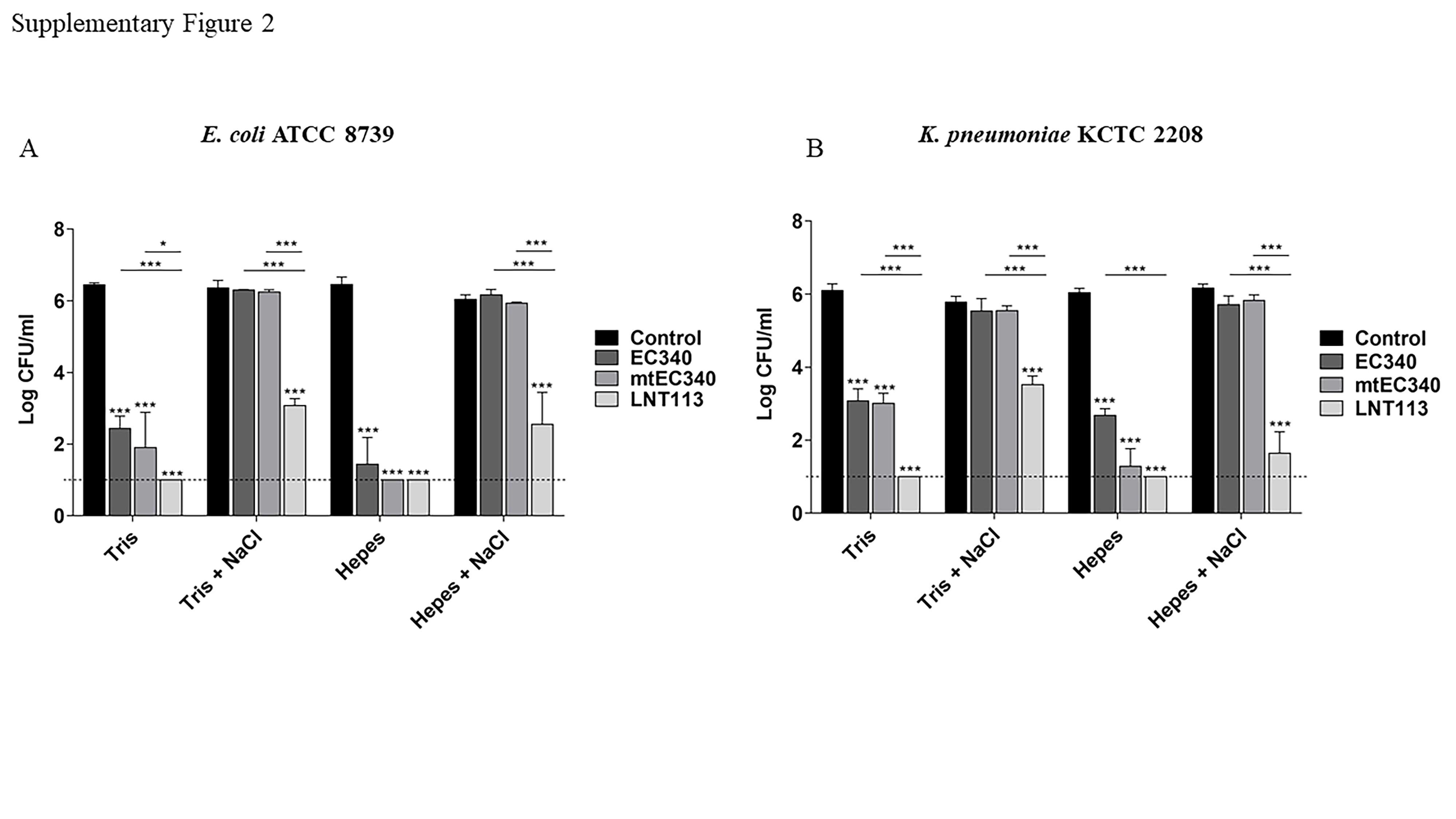

Supplement: Supplementary Figure 2 — Effect of turgor pressure and Tris in antibacterial activities of endolysins. Antibacterial activity of endolysins against E. coli ATCC8739 (A) and K. pneumoniae KCTC2208 (B). Exponentially grown bacterial cells were incubated with 2 μM of endolysins in two different buffers (20 mM Tris–HCl, pH 7.5 or 20 mM HEPES, pH7.5) in the presence or absence of 150 mM NaCl for 2 h at 37°C. Dotted line denotes the detection limit. In addition to t-tests, two-way ANOVA was performed and is shown as horizontal bars above vertical bars (*p < 0.05, **p < 0.01, ***p < 0.001). [file Image_2.TIF]
