# Supplementary material for: Combination Effect of Engineered Endolysin EC340 With Antibiotics
Source: Front Microbiol. 2022 Feb 15;13:821936. doi: 10.3389/fmicb.2022.821936 (PMC8886149; doi:10.3389/fmicb.2022.821936)
Supplement: Supplementary file 3 [file Table_1.DOCX]

**Supplementary Table 1. MICs of various antibiotics against 3 different drug resistant strains from CCARM**

| MIC (µg/ml)  of antibiotics | *E. coli* | | *K. pneumoniae* |
| --- | --- | --- | --- |
|  | CCARM 1A746 | CCARM 1B684 | CCARM 10143 |
| Ampicillin | ≥128 | ≥128 | ≥128 |
| Cephalothin | ≥128 | 64 | ≥128 |
| Ciprofloxacin | 128 | 64 | ≤0.25 |
| Gentamicin | ≥128 | 128 | ≥128 |
| Tetracycline | 128 | ≥128 | 2 |
| Cefotaxime | 128 | 0.25 | 16 |
| Trimethoprim-  sulfamethoxazole | ≥128 | - | ≥32 |
| Streptomycin | ≥128 | ≥128 | - |
| Norfloxacin | ≥128 | 32 | - |

Source: http://knrrb.ccarm-bio.or.kr

-: not determined
