# Supplementary material for: Combination Effect of Engineered Endolysin EC340 With Antibiotics
Source: Front Microbiol. 2022 Feb 15;13:821936. doi: 10.3389/fmicb.2022.821936 (PMC8886149; doi:10.3389/fmicb.2022.821936)
Supplement: Supplementary file 4 [file Table_2.DOCX]

**Supplementary Table 2. MIC (µg/ml) of 3 different endolysins in combination with colistin against various *E. coli* strains**

| ATCC8739 | | UPEC3150 | | FORC81 | | ATCC8739 | | ATCC8739 | |
| --- | --- | --- | --- | --- | --- | --- | --- | --- | --- |
| LNT113 | Colistin | LNT113 | Colistin | LNT113 | Colistin | EC340 | Colistin | mtEC340 | Colistin |
| 8 | 0 | 8 | 0 | 8 | 0 | >128 | 0 | >128 | 0 |
| 4 | 0.0625 | 4 | 0.0625 | 4 | 1 | 32 | 0.0625 | 32 | 0.125 |
| 2 | 0.5 | 2 | 0.5 | 2 | 4 | 16 | 0.125 | 16 | 0.125 |
| 1 | 1 | 1 | 2 | ≤0.25 | 8 | ≤2 | 0.25 | ≤2 | 0.25 |
| 0 | 2 | 0 | 4 | 0 | 16 | 0 | 2 | 0 | 2 |

ATCC8739, a type strain

UPEC3150, a UPEC strain

FORC81, a colistin-resistant strain (mcr-1)
